# Trali ou œdème pulmonaire lésionnel aigu post-transfusionnel: à propos d'un cas avec revue de la literature

**DOI:** 10.11604/pamj.2015.21.109.6435

**Published:** 2015-06-10

**Authors:** Issam Serghini, Youssef Nader, Youssef Qamouss, Mohamed Zoubir, Jaafar Salim Lalaoui, Mohamed Boughalem

**Affiliations:** 1 Pôle Anesthésie- Réanimation, Hôpital Militaire Avicenne, Faculté de Médecine et de Pharmacie, Université Cadi Ayyad, 40010 Marrakech, Maroc; 2 Pôle de Traumatologie Orthopédie, Hôpital Militaire Avicenne, Faculté de Médecine et de Pharmacie, Université Cadi Ayyad, 40010 Marrakech, Maroc

**Keywords:** Trali, réactions post transfusionnelles, œdème pulmonaire post-transfusionnel, Trali, post transfusion reactions, post-transfusion pulmonary edema

## Abstract

L’œdème pulmonaire lésionnel aigue transfusionnel est une complication classique de la transfusion de produits sanguins labiles, responsable de 17% des décès liés à la transfusion. Il s'agit d'un syndrome de détresse respiratoire aiguë post-transfusionnel qui se présente comme un œdème pulmonaire aigu non cardiogénique survenant dans les six heures suivant la mise en oeuvre d'une transfusion sanguine. Le mot Trali utilisé pour désigner cet œdème est l'acronyme de l'anglais « transfusion-related acute lung injury »; l'incidence des TRALI déclarés en France reste faible à cause de leurs méconnaissances, faute d'une définition précise tant au plan clinique qu'au plan étiologique. La sensibilisation des médecins à l'identification des TRALI et à la déclaration, notamment en réanimation, doit être poursuivie. Des données récentes ont souligné sa relative fréquence et des études cliniques et biologiques plus attentives ont contribué à une meilleure compréhension de ses mécanismes dont deux sont bien défini: un conflit immunologique d'une part, une activation des polynucléaires neutrophiles par des facteurs lipidiquesd'autre part. Il est également admis que le Trali ne se déclenche que dans un contexte favorisant dont le substratum commun pourrait essentiellement être une leucostase préalable. Le traitement du Trali est celui des œdèmes pulmonaires lésionnels, oxygénothérapie et assistance respiratoire. Nous rapportons un cas de Trali survenu à la suite de la transfusion de 08 concentrés de globules rouges chez un jeune homme de 26 ans.

## Introduction

L'oedème pulmonaire lésionnel aigu post-transfusionnel est connu depuis les années 1950 [1] où il fut identifié comme un œdème pulmonaire post-transfusionnel non cardiogénique. Il a fallu cependant attendre 1985 pour que le nom de Trali lui soit donné par Popovsky et Moore [2]. Néanmoins ce syndrome est resté méconnu et son incidence sous-évaluée. Dans la littérature anglo-saxonne, le TRALI a une incidence estimée à presque un pour 5000 produits sanguins labiles [3, 4]. Il représente désormais la première cause de décès post-transfusionnels aux États-Unis [5]. En France, son incidence semble être d'un cas pour 60 539 PSL transfusés [6] et ne serait que la troisième cause de mortalité post-transfusionnelle [7]. En 2010, le rapport national français d'hémovigilance suggère son implication dans 20% des décès liés à la transfusion [8]. L'incidence des TRALI est réévaluée après application stricte des critères de Toronto [9]. Deux mécanismes principaux sont évoqués: immunologique, lié à une réaction entre un anticorps du donneur et un antigène du receveur; et non immunologique, consécutif à une activation leucocytaire par des lipides ou des cytokines présents dans les produits transfusés [10]. Nous rapportons le cas d'un œdème pulmonaire transfusionnel pouvant entrer dans ce cadre.

## Patient et observation

Il s'agit d'un jeune soldat âgée de 26 ans, sans antécédent pathologique particulier, ayant sauté sur une mine anti personnelle et admis au service d'accueil des urgences du 5 éme Hôpital militaire de Guelmim, dans un tableau de choc hémorragique grave sur amputation partielle du pied gauche avec fracas osseux et lésions des parties molles de l'avant-pied. Avant l'admission au bloc opératoire, un remplissage vasculaire par 1 litre de cristalloïdes associé à une transfusion de 05 culots globulaires siso groupe iso rhésus ont été entamé vu le tableau clinique inquiétant du patient: paleur, conjonctives décolorées, froideur des extrémités, une hypotension artérielle (PA: 75/49) et sur le plan biologique une déglobulisation: Hb à 5 g/dl; au bloc opératoire sous Anesthesie génerale, l'amputation du membre a été réalisée avec myoplastie.

**Figure 1:**
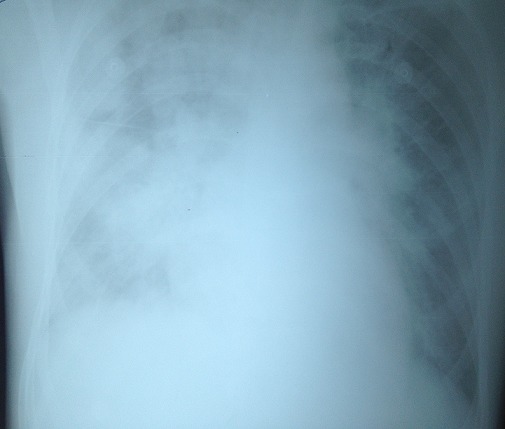
Alvéolo-interstitielles bilatérales sans élargissement de l'ombre cardiaque

Dans les suites opératoires, l'hémoglobinémie de contrôle était à 7g/dl, cliniquement bien tolérée. L'indication d'une transfusion par 03 unités de concentrés de globules rouges a été posée. Une heure après le début de la transfusion le patient a présenté une détresse respiratoire aiguë avec dyspnée, désaturation à 87%, cyanose et des frissons alors que des crépitants pulmonaires étaient auscultés aux deux bases et que s'installait une oligoanurie. L'administration de furosémide restait sans effet sur la diurèse et ne permettait pas d'améliorer l’état clinique du patient. Le reste de l'examen clinique retrouvait une tachycardie à 120 b/min, une hypotension artérielle à 80/50 mmHg et une pression veineuse centrale à 4 mmHg. Devant ce tableau clinique inquiétant le patient a été transféré en réanimation polyvalente. La gazométrie artérielle objectivait une hypoxie sévère avec une PaO2 à 53 mmHg en air ambiant La radiographie pulmonaire de face montrait des images alvéolo-interstitielles bilatérales sans élargissement de l'ombre cardiaque (Figure 1) et l’échocardiographie trans-thoracique était normale. En raison de cette dégradation clinique, le patient était intubé, ventilé et sédaté. Une hypotension à 60/30 mmHg nécessitait un remplissage vasculaire comportant deux litres de gélatine fluide modifiée et deux litres de cristalloïdes, ainsi que l'introduction d'adrénaline au pousse-seringue électrique (0,4 mg/h).

L’état clinique continuait à se détériorer avec l'extériorisation d'une très grande quantité d'oedème pulmonaire par la sonde d'intubation (aspiration en moins de deux heures d'environ 1 litre de sécrétions pulmonaires mousseuses). L'aggravation de l'hypoxémie et de l'hypotension était majeure. Ce choc restait complètement réfractaire à la thérapeutique (diurétiques et adrénaline) et entraînait le décès du patient en quelques heures. Les prélèvements à visée bactériologique étaient négatifs. Les tests immunologiques comprenant la recherche d'anticorps anti-HLA de classe I et II et d'anticorps leucoagglutinants, pratiqués sur les sérums de tous les donneurs des produits sanguins labiles impliqués, s'avérèrent normaux. Les anticorps n'ont pas pu être recherchés chez le receveur, faute de prélèvements sanguins adéquats.

## Discussion

L'installation d'un oedème aigu pulmonaire (OAP) lors d'une phase de remplissage vasculaire évoque en premier lieu une étiologie cardiaque. Cependant une autre étiologie doit être évoquée: l'oedème lésionnel post-transfusionnel appelé TRALI par les auteurs anglo-saxons. Le diagnostic de Trali est essentiellement clinique. Il survient dans la majorité des cas entre 30 minutes et deux heures et au plus tard dans les six heures après la mise en oeuvre d'une transfusion sanguine.

Dans sa forme typique, il associe une tachypnée avec cyanose, une toux, une extériorisation de sécrétions trachéales rmousseuses abondante riche en protéines, une élévation thermique supérieure ou égale à 1°C, une tachycardie inconstamment associée à une hypotension et des râles crépitants bilatéraux [11–13]. La radiographie thoracique retrouve généralement un syndrome interstitiel bilatéral, pouvant aller jusqu’à l'image de poumon blanc, cet aspect est évocateur d'un oedème aigu pulmonaire alors que l'index cardiothoracique est normal. La gazométrie montre une désaturation en oxygène du sang artériel, avec une saturation en oxygène inférieure ou égale à 90% et un rapport PAO2/FIO2 inférieure ou égale à 300 mmHg [11, 12]. La biologie comporte une neutropénie transitoire précoce, évocatrice mais inconstante [14, 15].

L'absence de défaillance cardiaque est la clé du diagnostic, confirmée par l’échocardiographie transthoracique avec doppler en permettant d’éviter un cathétérisme invasif et en objectivant une hypokinésie segmentaire, une cardiopathie sous-jacente et en mesurant de façon indirecte la fraction d’éjection ventriculaire gauche ainsi que les pressions de remplissages. La spécificité et la sensibilité de la BNP et NT-proBNP ne sont pas optimales dans ce contexte [16]. Les critéres de définition du TRALI retenue par l'Afssaps depuis 2006 sont ceux de la conférence de Toronto [17]. Celle-ci est clinique et radiologique basée sur les critères suivant: survenue brutale d'une détresse respiratoire aiguë; hypoxémie: PaO2/FiO2 < 300 (ALI) (Acute lung injury); PaO2/FiO2 < 200 (syndrome de détresse respiratoire aigu); SaO2 < 90% en air ambiant; infiltrats bilatéraux sur la radiographie pulmonaire de face; pas de d'argument pour une origine cardiogénique (PAPO < 18 mmHg par Swann Ganz ou arguments échographiques); absence d'ALI avant la transfusion; survenue dans les six heures suivant la transfusion; absence de relation temporelle avec un autre facteur de risque d'ALI.

Le traitement est symptomatique et repose sur une assistance respiratoire par oxygénothérapie au masque démarrée dés l'apparition des premiers signes cliniques, et la ventilation mécanique si nécessaire. Bien qu'elle n'ait pas été étudiée dans ce contexte étiologique, on peut penser que la ventilation non invasive conserve une indication identique à celle d'un oedème aigu pulmonaire standard (oedème aigu pulmonaire hypercapnique, détresse respiratoire, échec de traitement médical) [18]. Le recours au remplissage vasculaire et aux amines vasoactives est parfois indispensable. La corticothérapie n'a pas d'intérêt validé [19]. Les diurétiques pourraient être délétères du fait du risque d'hypovolémie [19]. Les produits sanguins labiles impliqués dans ce type d'accident sont: le sang total, les concentrés d’érythrocytes, le plasma frais congelé, les concentrés plaquettaires et plus rarement les immunoglobulines intraveineuses humaines polyvalentes. La physiopathologie du TRALI est basée sur la théorie du two hits model [17]. Cette hypothèse actuellement considérée comme la plus probable s'appuie sur l'existence d'un mécanisme receveur-dépendant et d'un mécanisme donneur-dépendant concomitant [17]. Le receveur est d'abord soumis à une agression (acte chirurgical, infection, polytraumatisme) aboutissant à une réponse systémique inflammatoire. Celle-ci se traduit par une libération accrue de cytokines et une activation de l'endothélium pulmonaire séquestrant ainsi de nombreux polynucléaires neutrophiles.

Cette cascade de mécanismes aboutit à une adhésion et une rigidité leucocytaire. Ensuite, l'activation et la dégranulation des polynucléaires séquestrés entraînent des lésions endothéliales et de la membrane alvéolaire [17]. Cette deuxième étape peut être d'origine: immunologique: par conflit antigène-anticorps granulocytaires (anticorps antiHLA I, anticorps antineutrophile, anticorps antiHLA II). Ces anticorps peuvent être recherchés chez le donneur et sont plus rarement identifiés chez le receveur; non immunologique: par accumulation de molécules lipidiques bioactives (lysophosphatidylcholines), lors de la consommation de produits sanguins cellulaires, par diminution de l'activité anti-oxydante liée à l'hypoxie ou par libération de sCD40L, médiateur pro inflammatoire présent dans les produits sanguins labiles; médiée par des facteurs de croissances endothéliaux vasculaires (VEGF) en particulier chez les patients neutropéniques

La confirmation biologique du TRALI doit être recherchée sous la forme d'anticorps antigranulocytaires ou anti-HLA classe I ou II chez le donneur et le receveur, en complément du bilan standard d'incident transfusionnel [20]. Si des anticorps sont retrouvés chez le donneur ou le receveur les groupes granulocytaires et des cross-matchs sont réalisés. La découverte d'un anticorps chez un des donneurs ne constitue pas, par elle-même, une preuve absolue et il est absolument nécessaire de contrôler la présence de l'antigène correspondant chez le receveur, ce qui n'est pas toujours possible compte tenu de la fréquente mortalité de ce syndrome. Dans ce cas on devra se contenter d'une présomption. En l'absence d'anticorps identifié on invoque le rôle de lipides activateurs dans le produit cellulaire transfusé [20].

Il manque à cette observation des éléments de certitude du diagnostic de TRALI. La recherche négative d'anticorps anti-HLA chez tous les donneurs de produits sanguins impliqués élimine l'hypothèse d'un TRALI par mécanisme immunologique. Une analyse des taux de lipides pro-inflammatoires au niveau des poches des produits sanguins aurait pu, si des taux élevés avaient été mis en évidence, rendre le diagnostic plus probable.

## Conclusion

La connaissance encore très incomplète des conditions de survenue du Trali, justifie qu'un effort particulier soit fait quant à son diagnostic, largement méconnu et au recueil des circonstances de survenue. Cela est pourtant un enjeu essentiel car mieux comprendre ces phénomènes, mieux les répertorier permettrait une évaluation des risques plus adaptée. Cette observation illustre donc les difficultés diagnostiques posées par la survenue d'un oedème pulmonaire au cours ou au décours immédiat d'une transfusion sanguine.
